# Long-Term Outcome with New Generation Prostheses in Patients Undergoing Transcatheter Aortic Valve Replacement

**DOI:** 10.3390/jcm10143102

**Published:** 2021-07-14

**Authors:** Alexander R. Tamm, Martin Geyer, Felix Kreidel, Lea Dausmann, Caroline Jablonski, Omar Hahad, Eberhard Schulz, Thomas Münzel, Ralph Stephan Von Bardeleben

**Affiliations:** 1 Department of Cardiology, Cardiology I, University Medical Center Mainz, Johannes Gutenberg University, Langenbeckstr. 1, 55131 Mainz, Germany; martin.geyer@unimedizin-mainz.de (M.G.); felix.kreidel@unimedizin-mainz.de (F.K.); dausmannl@gmail.com (L.D.); cjablons@students.uni-mainz.de (C.J.); omar.hahad@unimedizin-mainz.de (O.H.); tmuenzel@uni-mainz.de (T.M.); stephan.von_bardeleben@unimedizin-mainz.de (R.S.V.B.); 2 German Center for Cardiovascular Research (DZHK), Partner Site Rhine-Main, 55131 Mainz, Germany; 3 Department of Cardiology, Hospital Celle, Siemensplatz 4, 29223 Celle, Germany; eberhard.schulz@akh-celle.de

**Keywords:** aortic valve stenosis, TAVR, long-term outcomes, new-generation trans-catheter heart valves

## Abstract

The aim of this study was to compare patients with transcatheter aortic valve replacement (TAVR) receiving new generation prostheses SAPIEN 3 (S3, Edwards Lifesc.) and Evolut R (ER, Medtronic Inc.) in terms of periprocedural and long-term outcome. Our retrospective, single-center analysis included 359 consecutive patients with severe aortic stenosis who underwent TAVR with S3 or ER from 2014–2016 (mean age 82 ± 7 years, 47% male, mean EuroSCORE II 8.0 ± 8%, mean follow-up 3.8 years). Device Success was equal (S3 93.0% vs. ER 92.4%, *p* = 0.812). We report a 30-day mortality of 2.8% in the S3 group, and 2.1% in the ER group (*p* = 0.674). There was no difference in stroke, conversion to open surgery, vascular and bleeding complications or myocardial infarction. While prosthesis mean gradients were higher with S3 (12.0 mmHg vs. 8.2 mmHg, *p* < 0.001), there was a trend to less paravalvular regurgitation (PVR moderate or severe: 1% vs. 3.6%, *p* = 0.088). All-cause mortality up to 5 years did not show a difference (mean survival S3 3.5 ± 0.24 years, ER 3.3 ± 0.29 years, *p* = 0.895). Independent predictors of long-term mortality were impaired LVEF, chronic kidney injury, peripheral artery disease, malignant tumor and periprocedural stroke. New generation TAVR valves offer an excellent implant and outcome success rate. Long-term survival was independent of prostheses choice and mainly attributed to comorbidities and complications.

## 1. Introduction

Since the first transcatheter aortic valve replacement (TAVR) in 2002 [1] percutaneous therapy of aortic valve stenosis (AS) has evolved rapidly. Today, TAVR is the first option for older patients with AS at intermediate or high risk for surgery [2,3]. Constant development of bioprosthetic valves and delivery systems have reduced complication rates and improved outcomes over the years [4,5,6,7]. The 3rd generation Edwards SAPIEN 3 Valve (S3, Edwards Lifesciences Inc., Irvine, CA, USA) and the 2nd generation Medtronic Evolut R Valve (ER, Medtronic Inc. Minneapolis, MN, USA) are the most frequently used prostheses worldwide. There is a paucity of published data regarding long term outcomes in these new generation prostheses TAVR patients. We therefore present a single center analysis of TAVR with S3 and ER valves in comparison with focus on long-term follow up.

## 2. Materials and Methods

**Figure 1:**
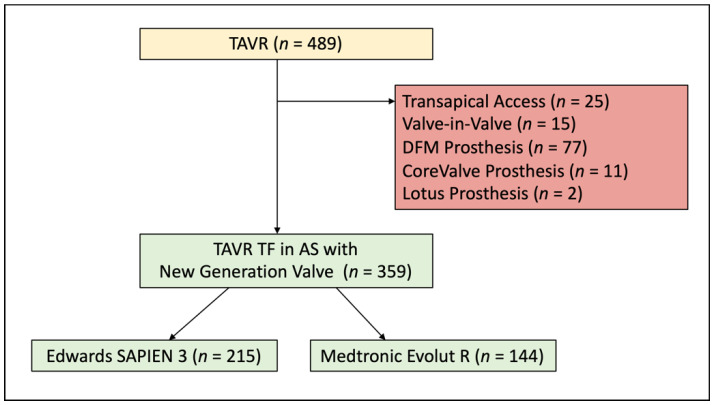
Patient Population. A total of 489 patients undergoing TAVR were screened for the analysis. Reasons for exclusion are displayed in the red box. TAVR = transcatheter aortic valve replacement; TF = transfemoral; AS = aortic stenosis; DFM = Direct Flow Medical.

Between June 2014 and May 2016, 489 consecutive patients underwent TAVR in the Heart Valve Center Mainz, Germany. We included 359 patients with severe degenerative aortic valve stenosis treated via transfemoral access using a new generation prosthesis (S3 or ER) in the study. Exclusion criteria were transapical access, valve-in-valve-procedure or the use of a non-ER or -S3 prosthesis type (Figure 1). The assignment to one of the valve platforms was chosen by the interventionalist regarding individual patient factors, which included calcification of the cusps, annulus and left ventricular outflow tract as well as possible need for future coronary intervention or beneficial femoral access. The TAVR procedure was performed according to the former standard protocol for transfemoral access that included general anesthesia and cross over safety technique. Pre- and post-dilatation were performed at the discretion of the interventionalist. The antithrombotic and antiplatelet regimen was standardized to lifetime Aspirin (or Warfarin in case of need for anticoagulation) and addition of Clopidogrel for 6 months.

Patient characteristics, imaging and procedural parameters were retrospectively recorded in a Microsoft Excel database (Microsoft Corporation, Redmond, WA, USA). The peri- and post-procedural outcomes of these patients were analyzed according to the updated criteria of the Valve Academic Research Consortium (VARC-2) [8]. In addition, VARC-2 composite end points of device success and early safety were calculated. The extent of calcification of the aortic valve commissures and annulus or LVOT was determined using separate four-step visual scales according to Marwan et al., and mean value was reported [9].

A follow-up on mortality status until August 2019 could be achieved in 92% of the patients through state civil registries and our University outpatient department. The study was approved by the local ethics committee (number 2019-14692).

All data is described as absolute numbers and percentage in case of nominal variables, as well as mean with standard deviation in case of metrical variables. Differences between patient groups were tested by Chi-squared test and Student’s *t*-test for nominal and metrical variables, respectively, if normally distributed, and with Mann-Whitney-U-Test if not normally distributed. Kaplan-Meier survival analysis was used for time dependent all-cause mortality. Comparison of the different groups was carried out with Cox proportional hazard models and Log-rank test. All analyses were performed with IBM SPSS Statistics version 24 (IBM Corporation, New York, NY, USA) and GraphPad PRISM version 8 (GraphPad Software, LLC., San Diego, CA, USA).

## 3. Results

The study population consisted of 359 patients with severe aortic stenosis who underwent transfemoral TAVR with a new generation prosthesis. The median follow-up period was 3.8 years (IQR 3.3 to 4.4 years, maximum follow-up in living patients 5.1 years). The SAPIEN 3 group included 215 patients, the Evolut R group 144 patients.

Baseline patient characteristics are displayed in Table 1. The mean patient age was 82 ± 7 years and 47% were men. The mean EuroSCORE II was 8.0 ± 8%; the mean STS Score PROM was 7.3 ± 9%. More patients in the S3 group were men (55% vs. 34%, *p* < 0.001), had a history of myocardial infarction (21% vs. 13%, *p* = 0.05) and were suffering from hypertension (92% vs. 82%, *p* = 0.006). S3 patients also had a lower mean left ventricular ejection fraction (50% vs. 55%, *p* < 0.001). In the ER group the prevalence of diabetes (28% vs. 38%, *p* = 0.041) and pulmonary hypertension (19% vs. 28%, *p* = 0.041) was higher, additionally these patients had a smaller calculated aortic valve area (0.8 cm^2^ vs. 0.7 cm^2^, *p* = 0.017).

Choice of prosthesis size differed significantly between both groups (Table 2). Whereas size distribution in the S3 group was balanced, in the ER group the 29 mm prosthesis was mainly implanted (76.4% of ER implantations). Balloon Pre-Dilatation was about equal in both groups (84.2% vs. 85.4%, *p* = 0.751), but need for post-dilatation was substantially higher in ER patients (0.5% vs. 18.8%, *p* < 0.001). Fluoroscopy time and volume of contrast did not differ between both groups.

Peri- and post-procedural outcome parameters are shown in Table 3. Device Success rates (VARC-2 composite endpoint) were about equal in both groups (93.0% vs. 92.4%, *p* = 0.812). We report a 30-day mortality of 2.8% in the S3 group and 2.1% in the ER group, respectively (*p* = 0.674). There was no difference in stroke rate, conversion to open heart surgery, major vascular complications, life-threatening or disabling bleeding or myocardial infarction. The VARC-2 composite endpoint of Early Safety at 30 days also did not differ significantly between both groups (11.2% vs. 7.6%, *p* = 0.270). Patients in the S3 group had a higher incidence of periprocedural acute kidney injury which did not reach statistical significance (2.3% vs. 0%, *p* = 0.065)

Implantation of a new permanent pacemaker (PPI) was lower in the S3 group (27.4% vs. 44.5%, *p* = 0.002). While prosthesis mean gradients were higher in the S3 group (12.0 mmHg vs. 8.2 mmHg, *p* < 0.001), there was a tendency to less paravalvular leaks (PVL ≥ 2: 1% vs. 3.6%, *p* = 0.088). Furthermore, there was early improvement of Left Ventricular Ejection Fraction, Mitral Regurgitation and Systolic Pulmonary Pressures at 30 days, which showed consistent values at one year follow up (Supplement Table S1).

**Figure 2:**
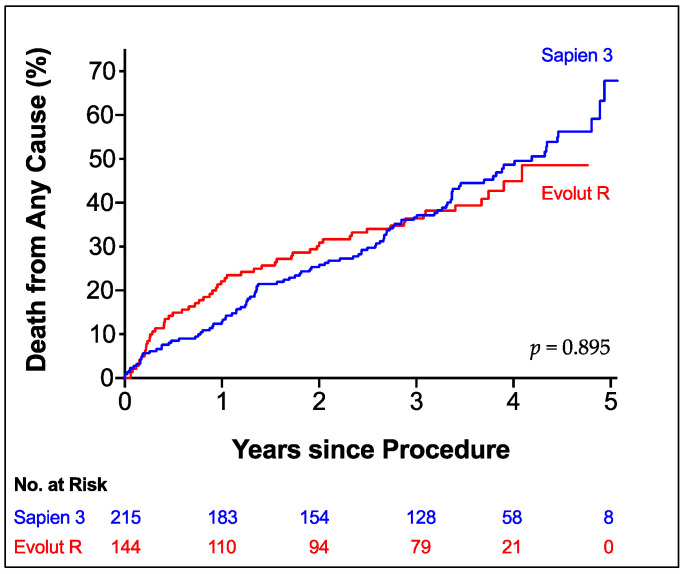
All-Cause Mortality. Kaplan-Meier Estimator of Death from any cause according to prosthesis type up to 5 years of follow-up. Edwards SAPIEN 3 (blue line); Medtronic Evolut R (red line).

**Figure 3:**
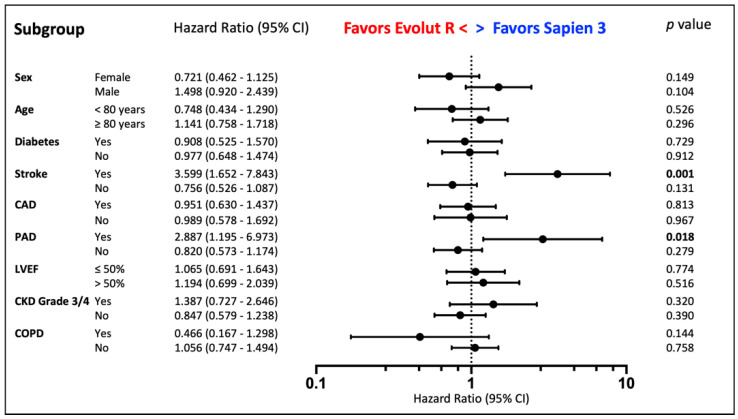
Prosthesis Choice. Subgroup Analysis by Cox Regression according to Prosthesis Choice. Stroke = History of Stroke, CAD = Coronary Artery Disease, PAD = Peripheral Artery Disease, CKD = Chronic Kidney Disease, LVEF = Left Ejection Fraction, COPD = Chronic Pulmonary Disease.

Recorded overall all-cause mortality was 16.3% at 1 year, 27.3% at 2 years and 37.5% at 3 years. Estimated mortality by Kaplan-Meier analysis (Figure 2) did not show a difference between both patient groups in long-term follow-up (mean survival S3 3.5 years, ER 3.3 years, *p* = 0.895). Subgroup analysis demonstrated a survival benefit for S3 patients with peripheral disease (HR for mortality ER vs. S3: 2.887, *p* = 0.018) or history of stroke (HR 3.599, *p* = 0.001). Additionally, male patients seemed to benefit from S3 (HR 1.498, *p* = 0.104) and female patients from ER implantation (HR 0.721, *p* = 0.149), although both did not reach statistical significance (Figure 3).

Univariate analysis (Supplement Table S2) showed a correlation between death of any cause and LVEF ≤ 50% (HR 1.65, *p* = 0.001), peripheral artery disease (HR 1.81, *p* = 0.011), chronic kidney injury (HR 1.49, *p* = 0.027) and history of cancer (HR 2.03, *p* < 0.001). In multivariate analysis (Supplement Table S2) independent predictors of all-cause mortality were LVEF ≤ 50% (HR 1.65, *p* = 0.005), peripheral artery disease (HR 2.30, *p* = 0.001), chronic kidney injury (HR 1.52, *p* = 0.042), COPD (HR 1.70, *p* = 0.043), history of cancer (HR 2.45, *p* < 0.001) and periprocedural stroke (HR 4.22, *p* = 0.007). However, the type of prosthesis was no predictor of mortality (HR 0.94, *p* = 0.732).

## 4. Discussion

With this study we present a comparison of the new-generation aortic valve prostheses Edwards SAPIEN 3 and Medtronic Evolut R concerning long-term as well as peri- and post-procedural outcomes. The analyzed cohort consisted of patients at intermediate to high surgical risk expressed through a EuroSCORE II of 8.0% and STS Score of 7.3% in mean, respectively. Device success in both groups was comparably good and is in line with previous studies using VARC-2 definitions [10,11]. Although ER patients needed significantly more post-dilatation after TAVR, fluoroscopy times and contrast use were about equal between both groups. These findings are supported by the literature and are constant with older and new generation valves [10,12].

Regarding periprocedural outcomes, both prostheses showed similar favorable results with very low incidences of stroke, vascular or bleeding complications as well as myocardial infarction or conversion to open heart surgery. Nevertheless, acute kidney injury stage 2 or higher was tendentially more often recorded in the S3 group. Finkelstein et al. describe similar observations, while other studies do not see differences between both prostheses [10,13,14]. Additionally, patients in the ER group had lower prosthetic mean gradients in the follow up echocardiography, which is a common finding in the literature [10,13]. An explanation should be the supra-annular design of the Evolut R platform as well as the bigger number of smaller prostheses in the S3 group (38.6% use of the 23 mm valve).

In general, we could show a very low incidence of relevant paravalvular regurgitation (moderate or severe PVL 1.9%), a complication that has been associated with worse long-term survival before [15]. These findings are comparable to the reported rates of moderate or severe PVL with new generation prostheses in the literature (S3 1.4–3.1%, ER 1.9–4.0%) [11,13,16,17]. Nevertheless, there was a tendency to less PVL in the S3 group (S3 1.0%, ER 3.6%, *p* = 0.088) which can be related to the newly developed outer skirt on the SAPIEN 3 prosthesis. Another confounder could be a selection tendency towards ER in very calcified annuli/LVOT that would lead to worse sealing. A further development of the Evolut platform with an added sealing skirt was not available at the time of patient inclusion (Evolut Pro, CE-Mark 2017).

The numbers of postinterventional need for permanent pacemaker seem high in comparison to similar studies (S3: 7.8–24.0%, ER: 16.4–25.0%), which could be explained by a low threshold for implantation in our institution at that time (including persisting left bundle branch block with AV-Block °I or bradyarrhythmia). However, the difference between both groups in favor of SAPIEN 3 (ER patients had 1.6-fold higher PPI rates compared to S3 patients) is comparable to the literature [10,11,16,18,19,20].

Despite treating a population at substantially increased risk, the 30-day mortality was very low at 2.5% overall with no relevant difference between both groups. Previous studies show comparable mortality data (S3: 0–3.1%, ER 1.9–3.4%) with transfemoral TAVR patients at lower to similar risk profile (EuroSCORE II 5.2–6.1%, STS Score 4.3–7.7%) [10,11,16,18,19,20].

Regarding survival there was no difference between S3 and ER patients with a mean survival of 3.5 and 3.3 years, respectively. This finding could also be confirmed in multivariate analysis (HR 0.937, *p* = 0.732). A recent work by Finkelstein et al. presented data with the same prostheses in low-risk patients showing similar mortality between both groups up to 3 years [13]. Vollenbroich et al. who studied long-term outcomes with the older platforms Edwards SAPIEN THV/XT and Medtronic CoreValve in a similar risk cohort could also show comparable all-cause mortality at 5 years between both patient groups (53.4% vs. 46.9%, *p* = 0.15) [21].

As in most studies concerning TAVR, long-term survival was mainly attributed to comorbidities such as reduced LVEF, peripheral artery disease, chronic kidney injury, advanced COPD or history of cancer. In addition, periprocedural stroke was the most important independent predictor of all-cause mortality up to five years. This finding is supported by a work from Levi et al. that showed a similar relevance of in-hospital stroke for long-term survival [22].

### Limitations of the Study

The present study has to be interpreted with regard to several limitations. First, it is a retrospective analysis that has inherent limitations and is open to bias. Second, the study was conducted at a single center, thus results might not be generally applicable. Third, the choice of the platform was not randomized but up to the decision of the implanter which could result in biased periprocedural outcome parameters. Since 34 mm ER was not on the market in the reported inclusion time, there might be a bias towards treating more patients with larger annuli with the S3 29 mm valve, which could explain the higher number of male patients treated in the SAPIEN group. At 5 years after implantation the number of patients that are included in the follow-up is low, thus the median follow-up time of 3.8 years could also be addressed as intermediate- to long-term follow-up.

## 5. Conclusions

Transcatheter aortic valve replacement (TAVR) has become a standard therapy in treating AS. Published data regarding long-term outcomes in patients with new generation TAVR prostheses (Edwards SAPIEN 3 Valve, Medtronic Evolut R Valve) are scarce.

We therefore present a large single center analysis of TAVR with SAPIEN 3 and Evolut R valves in comparison with focus on long-term follow up. Our single-center cohort data adds an industry-independent comparison between new-generation valves concerning periprocedural outcomes and long-term survival in a routine TAVR cohort. We demonstrated low procedural mortality and low paravalvular leakage rates in new generation TAVR. Mid- to long-term follow-up shows favorable similar survival in both prosthesis groups.

## Figures and Tables

**Table 1:** Baseline Patient Characteristics.

Characteristic	All Patients (*n* = 359)	SAPIEN 3 (*n* = 215)	Evolut R (*n* = 144)	*p*-Value
Age (Years)	81.9 ± 6.6	81.7 ± 6.6	82.2 ± 6.7	0.469
Male Sex-*n* (%)	167 (46.5)	118 (54.9)	49 (34)	<0.001
BMI (kg/m^2^)	27.1 ± 5.4	27.2 ± 5.2	27 ± 5.8	0.773
EuroSCORE II	8.0 ± 8.1	8.2 ± 8.2	7.8 ± 8.1	0.621
STS Score PROM	7.3 ± 8.9	7.3 ± 6.8	7.4 ± 5.0	0.092
NYHA Class ≥ III-*n* (%)	259 (72.1)	149 (69.3)	110 (76.4)	0.142
Coronary Artery Disease-*n* (%)	224 (62.4)	137 (63.7)	87 (60.4)	0.526
Previous Myocardial Infarction-*n* (%)	62 (17.3)	44 (20.5)	18 (12.5)	0.05
Previous PCI-*n* (%)	140 (39)	89 (41.4)	51 (35.4)	0.255
Previous CABG-*n* (%)	45 (12.5)	28 (13.0)	17 (11.8)	0.733
Previous Stroke or TIA-*n* (%)	60 (16.7)	42 (19.5)	18 (12.5)	0.08
Peripheral Art. Disease ≥ Grade II	33 (9.2)	17 (7.9)	16 (11.1)	0.303
Art. Hypertension-*n* (%)	314 (87.5)	196 (91.6)	118 (81.9)	0.006
Diabetes (%)	114 (31.7)	59 (27.9)	55 (38.2)	0.041
COPD ≥ Grade II (GOLD)-*n* (%)	37 (10.3)	23 (10.7)	14 (9.7)	0.766
GFR < 30 mL/min	62 (17.3)	34 (15.8)	28 (19.4)	0.372
History of Cancer	45 (12.5)	26 (12.1)	19 (13.2)	0.757
Atrial Fibrillation-*n* (%)	87 (24.2)	55 (25.9)	32 (22.4)	0.66
Permanent Pacemaker/ICD-*n* (%)	41 (11.4)	25 (11.6)	16 (11.1)	0.88
Pulm. Hypertension-*n* (%)	80 (22.3)	40 (18.6)	40 (27.8)	0.041
Left Ventricular Ejection Fraction (%)	51.5 ± 13.5	49.6 ± 14.4	54.5 ± 11.3	<0.001
Aortic Valve Area (cm^2^)	0.74 ± 0.2	0.80 ± 0.2	0.71 ± 0.2	0.017
Aortic Valve Peak Gradient (mmHg)	68.4 ± 25.7	67.5 ± 25.5	69.9 ± 26	0.367
Aortic Valve Mean Gradient (mmHg)	40.4 ± 16.8	39.9 ± 16.4	41.2 ± 17.2	0.493
Commissural Calcification (mean)	1.80 ± 0.7	1.80 ± 0.8	1.81 ± 0.7	0.941
Annular/LVOT Calcification (mean)	0.77 ± 0.9	0.70 ± 0.9	0.88 ± 1.0	0.077

BMI = Body Mass Index, EuroSCORE = European System for Cardiac Operative Risk Evaluation, STS = Society of Thoracic Surgeons, PROM = predicted risk of mortality, NYHA class = New York Heart Association Functional Classification of Heart Failure, PCI = Percutaneous Coronary Intervention, CABG = Coronary Artery Bypass Graft, TIA = Transient Ischemic Attack, COPD = Chronic Obstructive Pulmonary Disease, GFR = Glomerular filtration rate, ICD = Internal Cardioverter Defibrillator.

**Table 2:** Procedural Details.

	SAPIEN 3 (*n* = 215)	Evolut R (*n* = 144)	*p*-Value
Prosthesis Size			<0.001
23 mm-*n* (%)	83 (38.6)	2 (1.4)	
26 mm-*n* (%)	91 (42.3)	32 (22.2)	
29 mm-*n* (%)	41 (19.1)	110 (76.4)	
Balloon Pre-Dilatation	181 (84.2)	123 (85.4)	0.751
Balloon Post-Dilatation	1 (0.5)	27 (18.8)	<0.001
Fluoroscopy Time (min)	25.4 ± 11.4	24.5 ± 10.2	0.776
Contrast Volume (cc)	172.1 ± 61.1	157.5 ± 56.4	0.454

**Table 3:** Peri- and Post-procedural Outcome.

	All Patients (*n* = 359)	SAPIEN 3 (*n* = 215)	Evolut R (*n* = 144)	*p*-Value
30-Day Mortality	9 (2.5)	6 (2.8)	3 (2.1)	0.674
VARC-2 Device Success-*n* (%)	333 (92.8)	200 (93.0)	133 (92.4)	0.812
VARC-2 Early Safety at 30 days-*n* (%)	35 (9.7)	24 (11.2)	11 (7.6)	0.27
Conversion to Open Heart Surgery	3 (0.8)	2 (0.9)	1 (0.7)	0.810
Stroke-*n* (%)	8 (2.2)	6 (2.8)	2 (1.4)	0.378
Major Vascular Complication-*n* (%)	14 (3.9)	7 (3.3)	7 (4.9)	0.441
Life-Threatening or Disabling Bleeding-*n* (%)	6 (1.7)	4 (1.9)	2 (1.4)	0.733
Myocardial infarction-*n* (%)	1 (0.3)	1 (0.5)	0	0.412
Acute Kidney Injury ≥ 2-*n* (%)	5 (1.4)	5 (2.3)	0	0.065
Permanent Pacemaker Implantation-*n* (%)	109/318 (34.3)	52/190 (27.4)	57/128 (44.5)	0.002
Prosthesis Mean Gradient (mmHg)	10.5 ± 5.0	12.0 ± 4.4	8.2 ± 5.1	<0.001
Paravalvular Regurgitation ≥ 2-*n* (%)	7/348 (1.9)	2/209 (1.0)	5/139 (3.6)	0.088

VARC-2 = Valve Academic Research Consortium Updated Standardized Endpoint Definitions, Explanation of Composite Endpoints: Device Success = absence of procedural mortality, correct positioning of a single prosthetic heart valve and intended performance of prosthetic valve. Early Safety at 30 days = all-cause mortality, stroke, life-threatening bleeding, acute kidney injury ≥ 2, coronary artery obstruction requiring intervention, major vascular complication, valve-related dysfunction requiring repeat procedure.

## Data Availability

The datasets generated for this study are available on request to the corresponding author.

## Supplementary Materials

The following are available online at https://www.mdpi.com/article/10.3390/jcm10143102/s1, Table S1: Echocardiographic Parameters; Table S2: Predictors of All-Cause Mortality.

## References

[1] Cribier A., Eltchaninoff H., Bash A., Borenstein N., Tron C., Bauer F., Derumeaux G., Anselme F., Laborde F., Leon M.B. (2002). Percutaneous transcatheter implantation of an aortic valve prosthesis for calcific aortic stenosis: First human case description. Circulation.

[2] Baumgartner H., Falk V., Bax J.J., De Bonis M., Hamm C., Holm P.J., Iung B., Lancellotti P., Lansac E., Rodriguez Munoz D. (2017). ESC/EACTS guidelines for the management of valvular heart disease. Eur. Heart J..

[3] Nishimura R.A., Otto C.M., Bonow R.O., Carabello B.A., Erwin J.P., 3rd Fleisher L.A., Jneid H., Mack M.J., McLeod C.J., O’Gara P.T. (2017). 2017 AHA/ACC focused update of the 2014 AHA/ACC guideline for the management of patients with valvular heart disease: A report of the American College of Cardiology/American Heart Association Task Force on clinical practice guidelines. Circulation.

[4] Adams D.H., Popma J.J., Reardon M.J., Yakubov S.J., Coselli J.S., Deeb G.M., Gleason T.G., Buchbinder M., Hermiller J., Kleiman N.S. (2014). Transcatheter aortic-valve replacement with a self-expanding prosthesis. N. Engl. J. Med..

[5] Reardon M.J., Van Mieghem N.M., Popma J.J., Kleiman N.S., Sondergaard L., Mumtaz M., Adams D.H., Deeb G.M., Maini B., Gada H. (2017). Surgical or transcatheter aortic-valve replacement in intermediate-risk patients. N. Engl. J. Med..

[6] Leon M.B., Smith C.R., Mack M.J., Makkar R.R., Svensson L.G., Kodali S.K., Thourani V.H., Tuzcu E.M., Miller D.C., Herrmann H.C. (2016). Transcatheter or surgical aortic-valve replacement in intermediate-risk patients. N. Engl. J. Med..

[7] Smith C.R., Leon M.B., Mack M.J., Miller D.C., Moses J.W., Svensson L.G., Tuzcu E.M., Webb J.G., Fontana G.P., Makkar R.R. (2011). Transcatheter versus surgical aortic-valve replacement in high-risk patients. N. Engl. J. Med..

[8] Kappetein A.P., Head S.J., Genereux P., Piazza N., van Mieghem N.M., Blackstone E.H., Brott T.G., Cohen D.J., Cutlip D.E., van Es G.A. (2012). Updated standardized endpoint definitions for transcatheter aortic valve implantation: The Valve Academic Research Consortium-2 consensus document. J. Am. Coll. Cardiol..

[9] Marwan M., Achenbach S., Ensminger S.M., Pflederer T., Ropers D., Ludwig J., Weyand M., Daniel W.G., Arnold M. (2013). CT predictors of post-procedural aortic regurgitation in patients referred for transcatheter aortic valve implantation: An analysis of 105 patients. Int. J. Cardiovasc. Imaging.

[10] Ben-Shoshan J., Konigstein M., Zahler D., Margolis G., Chorin E., Steinvil A., Arbel Y., Aviram G., Granot Y., Barkagan M. (2017). Comparison of the Edwards SAPIEN S3 versus Medtronic Evolut-R devices for transcatheter aortic valve implantation. Am. J. Cardiol..

[11] Thiele H., Kurz T., Feistritzer H.J., Stachel G., Hartung P., Eitel I., Marquetand C., Nef H., Doerr O., Lauten A. (2020). Comparison of newer generation self-expandable vs. balloon-expandable valves in transcatheter aortic valve implantation: The randomized SOLVE-TAVI trial. Eur. Heart J..

[12] Abdel-Wahab M., Mehilli J., Frerker C., Neumann F.J., Kurz T., Tolg R., Zachow D., Guerra E., Massberg S., Schafer U. (2014). Comparison of balloon-expandable vs self-expandable valves in patients undergoing transcatheter aortic valve replacement: The CHOICE randomized clinical trial. JAMA.

[13] Finkelstein A., Steinvil A., Rozenbaum Z., Halkin A., Banai S., Barbash I., Guetta V., Segev A., Danenberg H., Orvin K. (2019). Efficacy and safety of new-generation transcatheter aortic valves: Insights from the Israeli transcatheter aortic valve replacement registry. Clin. Res. Cardiol..

[14] He C., Xiao L., Liu J. (2019). Safety and efficacy of self-expandable Evolut R vs. balloon-expandable SAPIEN 3 valves for transcatheter aortic valve implantation: A systematic review and meta-analysis. Exp. Ther. Med..

[15] Abdel-Wahab M., Zahn R., Horack M., Gerckens U., Schuler G., Sievert H., Eggebrecht H., Senges J., Richardt G. (2011). Aortic regurgitation after transcatheter aortic valve implantation: Incidence and early outcome. Results from the German transcatheter aortic valve interventions registry. Heart.

[16] Grube E., Van Mieghem N.M., Bleiziffer S., Modine T., Bosmans J., Manoharan G., Linke A., Scholtz W., Tchetche D., Finkelstein A. (2017). Clinical outcomes with a repositionable self-expanding transcatheter aortic valve prosthesis: The international FORWARD study. J. Am. Coll. Cardiol..

[17] Popma J.J., Adams D.H., Reardon M.J., Yakubov S.J., Kleiman N.S., Heimansohn D., Hermiller J., Hughes G.C., Harrison J.K., Coselli J. (2014). Transcatheter aortic valve replacement using a self-expanding bioprosthesis in patients with severe aortic stenosis at extreme risk for surgery. J. Am. Coll. Cardiol..

[18] Popma J.J., Reardon M.J., Khabbaz K., Harrison J.K., Hughes G.C., Kodali S., George I., Deeb G.M., Chetcuti S., Kipperman R. (2017). Early clinical outcomes after transcatheter aortic valve replacement using a novel self-expanding bioprosthesis in patients with severe aortic stenosis who are suboptimal for surgery: Results of the Evolut R U.S. study. JACC Cardiovasc. Interv..

[19] Webb J., Gerosa G., Lefevre T., Leipsic J., Spence M., Thomas M., Thielmann M., Treede H., Wendler O., Walther T. (2014). Multicenter evaluation of a next-generation balloon-expandable transcatheter aortic valve. J. Am. Coll. Cardiol..

[20] Vlastra W., Chandrasekhar J., Munoz-Garcia A.J., Tchetche D., de Brito F.S., Barbanti M., Kornowski R., Latib A., D’Onofrio A., Ribichini F. (2019). Comparison of balloon-expandable vs. self-expandable valves in patients undergoing transfemoral transcatheter aortic valve implantation: From the CENTER-collaboration. Eur. Heart J..

[21] Vollenbroich R., Wenaweser P., Macht A., Stortecky S., Praz F., Rothenbuhler M., Roost E., Hunziker L., Raber L., Windecker S. (2019). Long-term outcomes with balloon-expandable and self-expandable prostheses in patients undergoing transfemoral transcatheter aortic valve implantation for severe aortic stenosis. Int. J. Cardiol..

[22] Levi A., Landes U., Assali A.R., Orvin K., Sharony R., Vaknin-Assa H., Hamdan A., Shapira Y., Schwartzenberg S., Codner P. (2017). Long-term outcomes of 560 consecutive patients treated with transcatheter aortic valve implantation and propensity score-matched analysis of early- versus new-generation valves. Am. J. Cardiol..

