# Supplementary material for: Long-Term Outcome with New Generation Prostheses in Patients Undergoing Transcatheter Aortic Valve Replacement
Source: J Clin Med. 2021 Jul 14;10(14):3102. doi: 10.3390/jcm10143102 (PMC8303957; doi:10.3390/jcm10143102)
Supplement: Supplementary file 1 [file jcm-10-03102-s001.zip › jcm-1268166-supplementary.pdf]

Table S1. Echocardiographic Parameters.

		All Patients (N = 359)	SAPIEN 3 (N = 215)	Evolut (N = 144)	*p*-Value
Aortic Valve Area (cm2)	baseline	**0.74** ± 0.2	**0.76** ± 0.2	**0.71** ± 0.2	**0.017**
	30 days	**1.57** ± 0.4	**1.49** ± 0.4	**1.71** ± 0.4	**<0.001**
	1 year	**1.53** ± 0.5	**1.44** ± 0.5	**1.70** ± 0.5	**0.002**
Peak Gradient (mmHg)	baseline	68.4 ± 25.7	67.5 ± 25.5	69.9 ± 26	0.404
	30 days	18.2 ± 8.0	21.6 ± 7.6	12.8 ± 5.1	**<0.001**
	1 year	18.2 ± 8.0	21.6 ± 9.0	13.5 ± 4.9	**<0.001**
Mean Gradient (mmHg)	baseline	40.4 ± 16.8	39.9 ± 16.4	41.2 ± 17.2	0.487
	30 days	9.4 ± 4.1	11.2 ± 4.0	6.6 ± 2.5	**<0.001**
	1 year	9.4 ± 4.1	11.6 ± 5.4	6.8 ± 2.4	**<0.001**
Aortic Regurgitation / PVR ≥ 2 - n (%)	baseline	56 **(15.6)**	33 **(15.4)**	23 **(16.0)**	0.888
	30 days	7/346 **(2.0)**	2/207 **(1.0)**	5/139 **(3.6)**	0.088
	1 year	5/147 **(3.4)**	3/94 **(3.2)**	2/53 **(3.8)**	0.969
LVEF (%)	baseline	51.5 ± 13.5	49.6 ± 14.4	54.5 ± 11.3	**<0.001**
	30 days	55.9 ± 11.2	54.7 ± 11.7	57.7 ± 9.9	**0.045**
	1 year	55.9 ± 11.2	57.1 ± 11.3	55.9 ± 10.0	0.523
Systolic PAP (mmHg)	baseline	37.7 ± 14.8	36.1 ± 14.2	40.2 ± 15.2	**0.011**
	30 days	33.8 ± 10.8	32.8 ± 10.0	35.5 ± 11.9	0.074
	1 year	33.8 ± 10.8	34.7 ± 13.2	35.8 ± 11.7	0.651
Mitral Regurgitation ≥ 2 - n (%)	baseline	123 **(34.3)**	72 **(33.5)**	51 **(35.4)**	0.706
	30 days	48/187 **(20.4)**	29/116 **(20.0)**	19/71 **(21.1)**	0.837
	1 year	36/111 **(24.5)**	22/72 **(23.4)**	14/39 **(26.4)**	0.684
Tricuspid Regurgitation ≥ 2 - n (%)	baseline	92 **(25.8)**	47 **(22.0)**	45 **(31.5)**	**0.044**
	30 days	48/186 **(20.5)**	28/116 **(19.4)**	20/70 **(22.2)**	0.609
	1 year	36/111 **(24.5)**	19/75 **(20.2)**	17/36 **(32.1)**	0.108

LVEF - Left Ventricular Ejection Fraction, PAP - Pulmonary Artery Pressure

Table S2. Predictors of All-Cause Mortality.

	Univariate		Multivariate	
	**Hazard Ratio (CI)**	***p*-Value**	**Hazard Ratio (CI)**	***p*-Value**
Prosthesis (Sapien 3 vs. Evolut R)	1.034 (0.746–1.433)	0.843	0.937 (0.646–1.359)	0.732
Age	1.023 (0.998–1.049)	0.073	1.026 (0.995–1.058)	0.095
Sex (Male)	0.900 (0.661–1.226)	0.505	0.857 (0.598–1.227)	0.400
Coronary Artery Disease	1.125 (0.816–1.549)	0.472	1.048 (0.729–1.506)	0.799
Prior Coronary Bypass	0.831 (0.509–1.356)	0.459	0.664 (0.386–1.141)	0.138
Left Ventricular Ejection Fraction ≤ 50%	**1.649 (1.211–2.244)**	**0.001**	**1.649 (1.166–2.330)**	**0.005**
Diabetes	1.114 (0.802–1.547)	0.521	1.165 (0.807–1.681)	0.416
Peripheral Artery Disease	**1.814 (1.146–2.871)**	**0.011**	**2.298 (1.382–3.820)**	**0.001**
Chronic Kidney Injury (GFR ≤ 60ml/min)	**1.494 (1.048–2.131)**	**0.027**	**1.516 (1.015–2.265)**	**0.042**
COPD ≥ Grade II (GOLD)	1.273 (0.796–2.035)	0.313	**1.704 (1.016–2.856)**	**0.043**
History of Cancer	**2.025 (1.379–2.973)**	**<0.001**	**2.448 (1.638–3.660)**	**<0.001**
Stroke In-Hospital	1.572 (0.581–4.253)	0.373	**4.216 (1.486–11.960)**	**0.007**
Major Vascular Complication	1.700 (0.867–3.332)	0.122	1.398 (0.544–3.593)	0.487
Life-Threatening or Disabling Bleeding	2.175 (0.805–5.876)	0.126	1.547 (0.381–6.272)	0.542
Permanent Pacemaker Implantation	1.131 (0.814–1.571)	0.463	1.167 (0.822–1.658)	0.387
Paravalvular Regurgitation ≥ 2	1.453 (0.538–3.922)	0.461	1.462 (0.475–4.495)	0.508

GFR = Glomerular filtration rate, COPD = Chronic Obstructive Pulmonary Disease
